# Acute Mono-Arthritis Activates the Neurohypophysial System and Hypothalamo-Pituitary Adrenal Axis in Rats

**DOI:** 10.3389/fendo.2020.00043

**Published:** 2020-02-11

**Authors:** Haruki Nishimura, Makoto Kawasaki, Takanori Matsuura, Hitoshi Suzuki, Yasuhito Motojima, Kazuhiko Baba, Hideo Ohnishi, Yoshiaki Yamanaka, Teruaki Fujitani, Mitsuhiro Yoshimura, Takashi Maruyama, Hiromichi Ueno, Satomi Sonoda, Kazuaki Nishimura, Kentarou Tanaka, Kenya Sanada, Tatsushi Onaka, Yoichi Ueta, Akinori Sakai

**Affiliations:** ^1^Department of Orthopaedics Surgery, School of Medicine, University of Occupational and Environmental Health, Kitakyushu, Japan; ^2^Department of Physiology, School of Medicine, University of Occupational and Environmental Health, Kitakyushu, Japan; ^3^Division of Brain and Neurophysiology, Department of Physiology, Jichi Medical University, Shimotsuke, Japan

**Keywords:** oxytocin, arginine vasopressin, corticotropin-releasing hormone, hypothalamus, acute mono-arthritis, nociceptive pain, hypothalamic-pituitary-adrenal axis

## Abstract

Various types of acute/chronic nociceptive stimuli cause neuroendocrine responses such as activation of the hypothalamo-neurohypophysial [oxytocin (OXT) and arginine vasopressin (AVP)] system and hypothalamo-pituitary adrenal (HPA) axis. Chronic multiple-arthritis activates the OXT/AVP system, but the effects of acute mono-arthritis on the OXT/AVP system in the same animals has not been simultaneously evaluated. Further, AVP, not corticotropin-releasing hormone (CRH), predominantly activates the HPA axis in chronic multiple-arthritis, but the participation of AVP in HPA axis activation in acute mono-arthritis remains unknown. Therefore, we aimed to simultaneously evaluate the effects of acute mono-arthritis on the activity of the OXT/AVP system and the HPA axis. In the present study, we used an acute mono-arthritic model induced by intra-articular injection of carrageenan in a single knee joint of adult male Wistar rats. Acute mono-arthritis was confirmed by a significant increase in knee diameter in the carrageenan-injected knee and a significant decrease in the mechanical nociceptive threshold in the ipsilateral hind paw. Immunohistochemical analysis revealed that the number of Fos-immunoreactive (ir) cells in the ipsilateral lamina I–II of the dorsal horn was significantly increased, and the percentage of OXT-ir and AVP-ir neurons expressing Fos-ir in both sides of the supraoptic (SON) and paraventricular nuclei (PVN) was increased in acute mono-arthritic rats. *in situ* hybridization histochemistry revealed that levels of OXT mRNA and AVP hnRNA in the SON and PVN, CRH mRNA in the PVN, and proopiomelanocortin mRNA in the anterior pituitary were also significantly increased in acute mono-arthritic rats. Further, plasma OXT, AVP, and corticosterone levels were significantly increased in acute mono-arthritic rats. These results suggest that acute mono-arthritis activates ipsilateral nociceptive afferent pathways at the spinal level and causes simultaneous and integrative activation of the OXT/AVP system. In addition, the HPA axis is activated by both AVP and CRH in acute mono-arthritis with a distinct pattern compared to that in chronic multiple-arthritis.

## Introduction

The hypothalamo-neurohypophysial system, comprising oxytocin (OXT) and arginine vasopressin (AVP), regulates delivery, lactation, and homeostasis of plasma osmolality and water balance. Recent studies suggest that the OXT/AVP system and hypothalamo-pituitary adrenal (HPA) axis are activated by acute/chronic nociceptive stimuli and are involved in the modulation of nociceptive afferent pathways (1–6). Previous studies including our own (7–10) used several acute/chronic nociceptive models such as the formalin test and adjuvant arthritis model in rats to examine the effects of acute/chronic nociceptive stimuli on the OXT/AVP system. Formalin injection induces tissue injury and tonic pain (11). Although the formalin test is used as a method to evaluate animal behavior in the context of tonic pain (11–13), it does not truly model any clinical condition. Adjuvant arthritis is a model of poly-arthritis induced by immunogenic adjuvants (complete Freund's adjuvant) (14, 15). Our previous studies with the adjuvant arthritis model showed that chronic multiple-arthritis activates the OXT/AVP system separately (7, 10). Further, AVP, not corticotropin-releasing hormone (CRH), predominantly activates the HPA axis in chronic multiple-arthritis. However, the systemic nature of this arthritis may affect the overall condition and well-being of the animals and may confound pain assessment; therefore, the knee joint is not the primary target and area of interest in this model (16). In contrast, the acute inflammatory phase of knee osteoarthritis is mimicked by the carrageenan-induced knee joint arthritis model (17–19). To assess the involvement of the OXT/AVP system and HPA axis in common clinical conditions such as knee arthritis, carrageenan-induced knee arthritis in rats was used as a more ethologically valid model. Therefore, in the present study, we decided to assess the effects of (only) arthritis without any systemic factors using the acute mono-arthritis model induced by carrageenan on the activity of OXT/AVP system as well as the HPA axis in the same animals simultaneously. Acute mono-arthritis was induced by an injection of carrageenan into a single knee in rats. Carrageenan, a sulfated mucopolysaccharide extracted from seaweeds, is commonly used to induce acute mono-arthritic nociception in rodent studies (17, 20–22). Several studies have reported that OXT decreased carrageenan-induced inflammation and hyperalgesia (23, 24). Thus, it is assumed that OXT also modulates carrageenan-induced acute mono-arthritis.

## Materials and Methods

### Animals

Male Wistar rats (aged 7 weeks, weighing 240–320 g; *n* = 105) were purchased from Clea Japan, Inc. (Tokyo, Japan) and maintained as described previously (25). The rats were housed in cages and handled every day for at least 7 days before the start of the experiments. All rats were housed in groups of three per plastic cage in an air-conditioned room (22–25°C) on a 12:12-h light/dark cycle (lights on at 0700 h) with food and drinking water available *ad libitum* throughout the experiments. All experiments were performed in strict accordance with the Guiding Principles for the Care and Use of Animals in the Field of Physiological Sciences as issued by the Physiological Society of Japan and approved by the Ethics Committee of Animal Care and Experimentation of the University of Occupational and Environmental Health, Japan.

### Induction of Knee Arthritis With Carrageenan

The rats were divided randomly into three groups (*n* = 5–7 per group and experiment). In group 1 (Control), rats were only anesthetized without any intra-articular (IA) injections. In group 2 (Saline), rats received an IA injection of 0.1 mL of 0.9% NaCl. In group 3 (Carrageenan), an IA injection of 0.1 mL of 3% λ-carrageenan (Sigma, St. Louis, MO) in 0.9% NaCl was administered. IA injections of 0.9% NaCl or 3% λ-carrageenan were administered into the right hind knee joint using 25-gauge injection needles after anesthesia induction via inhalation of sevoflurane for 2–3 min in a glass chamber, according to a method published previously (26). Total 7 sets of experimental series were used for all experiments. One set of animals was used for measurement of joint swelling and the other one set was used for measurement of mechanical nociceptive threshold. Two sets were used for fluorescent immunohistochemistry (FIHC) at 3 or 12 h after IA injection and three sets were used for *in situ* hybridization histochemistry (ISH) and measurement of plasma concentrations at 2, 6, or 12 h after IA injection, respectively.

### Measurement of Joint Swelling

To assess joint swelling induced by carrageenan IA injections, the diameters of the right and left knee joints were measured using digital calipers before the IA injection as a baseline (BL) and at 3, 6, and 12 h after the IA injection on the same day with the 1st set of experimental series (*n* = 5–6 per group). The knee joint diameter was defined as the distance between the lateral and medial collateral ligament regions. This assessment procedure has been performed and published previously (27). The changes in knee diameter for each animal were calculated, and the results were averaged for each group at each evaluation time.

### Measurement of Mechanical Nociceptive Threshold

The mechanical nociceptive threshold was evaluated with the manual von Frey test using calibrated von Frey filaments (North Coast Medical, Gilroy, CA). Repetitive measurements were performed on the same animal as per the method reported by Shir et al. (28). Measurements were taken before the IA injection as a baseline (BL), and 3, 6, and 12 h after the IA injection with the 2nd set of experimental series (*n* = 6 per group). The rats adjusted to the experimental conditions for at least 30 min in an acrylic cage on an elevated mesh floor before the test. Mechanical stimulation to the plantar surface of the ipsilateral foot was performed using filaments ranging from 0.25 to 20.0 g. This repetitive stimulus was applied five times at a frequency of two stimuli per second, and the weakest force (g) to induce a paw withdrawal was regarded as the threshold. The average mechanical nociceptive threshold for each group at each evaluation time was calculated.

### Fluorescent Immunohistochemistry

#### Tissue Preparation

Tissue preparation was performed as described previously (29, 30). With the 3rd and 4th sets of experimental series, the rats were deeply anesthetized by intraperitoneal (i.p.) administration of a combination of anesthetics: 0.3 mg/kg of medetomidine, 4.0 mg/kg of midazolam, and 5.0 mg/kg of butorphanol at 3 or 12 h after the IA injection, respectively. The rats were perfused transcardially with 0.1 M phosphate buffer (PB) (pH 7.4) containing heparin (1,000 U/L), followed by 4% paraformaldehyde (PFA) in 0.1 M PB. The brains and spines were carefully removed, and the brains were divided into three blocks that included the hypothalamus.

The brains and spines were post-fixed with 4% PFA in 0.1 M PB for 48 h at 4°C. The tissues were then cryoprotected in 20% sucrose in 0.1 M PB for 48 h at 4°C. Fixed tissues were then cut into 30 μm sections with a microtome (Komatsu Electronics Co., Ltd., Hiratsuka, Japan). Brain sections including the hypothalamus and spinal sections including the lumbar segments (L) 3–5 were collected and stored in 0.1 M phosphate-buffered saline (PBS) at 4°C.

#### Fos Labeling on Spinal Cord Sections

For FIHC of Fos, spinal sections including L3–5 were incubated for 3 days at 4°C with a rabbit polyclonal c-Fos antibody (sc-52; Santa Cruz Biotechnology, Dallas, TX, USA; 1:1,000) in PBS containing 0.3% Triton X-100 (PBST). After washing three times in 0.1 M PBS for 30 min in total, the sections were incubated overnight at 4°C with secondary antibodies (goat Alexa Fluor 586-conjugated anti-rabbit IgG antibody; Molecular Probes, OR, USA; 1:1,000) in PBST. Sections were washed three times in 0.1 M PBS for 30 min in total.

The method for preparing the tissue specimens with stained sections and the analysis of specimens with microscopy were performed as described previously (31). The sections were mounted on glass slides and cover-slipped using Vectashield (Vector Laboratories Co. Ltd., CA, USA). The sections for L4 were examined by fluorescence microscopy (ECLIPSEE 600; Nikon Corp., Tokyo, Japan) with an RFP filter (Nikon Corp.). The sections for L4 were determined based on Figure 116 of the Rat Brain in Stereotaxic Coordinates atlas by Paxinos and Watson (32), and the images were obtained using a digital camera (DS-L2, DS-Fi1; Nikon Corp.). Fos-immunoreactive (ir) cells in lamina I and II of both ipsilateral and contralateral L4 spinal dorsal horns were counted manually with each captured image, as described previously (31, 33–36). Three sections of L4 were counted for each animal, and the results were averaged for each group at each evaluation time (*n* = 5–6 per group and evaluation time).

#### Fos Colocalization With OXT/AVP

For FIHC of Fos and OXT, brain sections including the hypothalamus were incubated for 3 days at 4°C with a goat polyclonal c-Fos antibody (sc-52G; Santa Cruz Biotechnology, Dallas, TX, USA; 1:500) and rabbit polyclonal OXT antibody (AB911; Merck Corp. Darmstadt, Germany; 1:10,000) in PBST with 5% normal donkey serum. After washing three times in 0.1 M PBS for 30 min in total, the sections were incubated overnight at 4°C with secondary antibodies (For Fos: donkey Alexa Fluor 586-conjugated anti-goat IgG antibody, Molecular Probes, OR, USA at 1:500; for OXT: donkey Alexa Fluor 488-conjugated anti-rabbit IgG antibody; Molecular Probes, OR, USA; 1:500) in PBST with 5% normal donkey serum. Sections were then washed three times in 0.1 M PBS for a total of 30 min.

For FIHC of Fos and AVP, brain sections including the hypothalamus were incubated for 3 days at 4°C with a goat polyclonal c-Fos antibody (sc-52G; Santa Cruz Biotechnology, Dallas, TX, USA; 1:500) and a rabbit polyclonal AVP antibody (Lot.1004001, Immunostar Inc., WI, USA; 1:10,000) in PBST with 5% normal donkey serum. After washing three times in 0.1 M PBS for a total of 30 min, the sections were incubated overnight at 4°C with secondary antibodies (for Fos: donkey Alexa Fluor 586-conjugated anti-goat IgG antibody, Molecular Probes, OR, USA, 1:500; for AVP: donkey Alexa Fluor 488-conjugated anti-rabbit IgG antibody, Molecular Probes, OR, USA; 1:500) in PBST and 5% normal donkey serum. Sections were washed three times in 0.1 M PBS for a total of 30 min.

The method for preparing the tissue specimens with stained sections and the analysis of specimens with microscopy were performed as described previously (7, 8, 31). The sections were mounted on glass slides and cover-slipped using Vectashield (Vector Laboratories Co. Ltd., CA, USA). Sections including the SON and PVN were examined by fluorescence microscopy (ECLIPSEE 600; Nikon Corp., Tokyo, Japan) with RFP and GFP filters (Nikon Corp.). Sections including the SON and PVN were determined based on Figure 25 of the Rat Brain in Stereotaxic Coordinates atlas by Paxinos and Watson (32). The images were obtained with a digital camera (DS-L2, DS-Fi1; Nikon Corp.).

To assess the percentage of Fos induction in OXT and AVP neurons, Fos-immunoreactive (ir) cells (appearing as round and red elements), OXT or AVP-ir cells (appearing as green cytoplasmic staining), and double-positive cells (appearing as green cytoplasmic staining containing red nuclei in merged images) were counted manually for each captured image. We counted two cross sections of each nucleus (for OXT: SON, mPVN, pPVN, and the anterior part of pPVN [apPVN]; for AVP: SON, mPVN, and pPVN) for each animal, and the results were averaged for each group at each evaluation time (*n* = 5–6 per group and evaluation time). In addition, the analyzed areas (for OXT: SON, mPVN, pPVN, and apPVN; for AVP: SON, mPVN, and pPVN) were measured with NIS-Elements software (Nikon Corporation, Tokyo, Japan).

### *In situ* Hybridization Histochemistry for *OXT, CRH*, and *POMC* mRNA, and *AVP* hnRNA

With the 5, 6, and 7th sets of experimental series, the rats were decapitated at 2, 6, or 12 h after IA injection, respectively. The brains and hypophyses were rapidly removed and placed onto a glass plate on dry ice and stored at −80°C until they were used for ISH for *OXT, CRH*, and proopiomelanocortin (*POMC*) mRNA, and *AVP* hnRNA.

Tissue preparation and ISH procedures were performed as described previously (37). Frozen brain coronal sections (12 μm thickness) were cut at −20°C, thawed, and mounted onto gelatin/chrome alum-coated slides. The sections including SON were used for ISH of *OXT* mRNA and *AVP* hnRNA. Sections including the PVN were used for ISH of *OXT* and *CRH* mRNA, and *AVP* hnRNA. Sections including the SON and PVN were determined based on Figure 25 of the Rat Brain in Stereotaxic Coordinates atlas by Paxinos and Watson (32). Sections were assessed with dark-field microscopy to select the sections that corresponded to those in the atlas. Further, frozen pituitary axial sections (12 μm thickness) were cut in the same way, and sections including the anterior pituitary were used for ISH of *POMC* mRNA. Two, two, and six sections containing the SON, PVN, and anterior pituitary, respectively, were used from each rat to determine the autoradiography density. ^35^S 3′-end-labeled deoxyoligonucleotides that were complementary to the transcripts encoding *OXT, AVP, CRH*, and *POMC* were used (OXT probe sequence, 5′-CTC GGA GAA GGC AGA CTC AGG GTC GCA GGC-3′; AVP probe sequence, 5′-GCA CTG TCA GCA GCC CTG AAC GGA CCA CAG TGG TAC-3′; CRH probe sequence, 5′-CAG TTT CCT GTT GCT GTG AGC TTG CTG AGC TAA CTG CTC TGC CCT GGC-3′; and POMC probe sequence, 5′-TGG CTG CTC TCC AGG CAC CAGCTC CAC ACA TCT ATG GAG G-3′). The probe was 3′-end labeled with terminal deoxynucleotidyl transferase and [^35^S] deoxy-ATP. Autoradiographic images for two sections containing the SON and PVN for *OXT* and *CRH* mRNA, as well as *AVP* hnRNA; and six sections containing the AP for *POMC* mRNA were taken by a charge-coupled device camera (DAGE-MTI, Inc., IN, USA). These images were analyzed for mean gene expression levels for each animal using ImageJ software (National Institutes of Health, MD, USA). For *OXT* mRNA and *AVP* hnRNA, gene expression levels per unit area in the SON, mPVN, and pPVN were analyzed. Regarding *CRH* mRNA, areas of the pPVN were analyzed. For *POMC* mRNA, areas of the AP were analyzed. To determine the area for evaluation of *AVP* hnRNA expression levels in the pPVN, serial brain sections containing the pPVN were used for analyzing *AVP* hnRNA and *CRH* mRNA, and the area expressing *CRH* mRNA was determined as the pPVN. This area was traced and copied onto the images of the sections for AVP containing the PVN, as described previously (9). Finally, the averages of those gene expression levels were calculated for each group at each evaluation time (*n* = 6–7 per group and evaluation time).

### Measurement of Plasma OXT, AVP, and Corticosterone

The trunk blood of rats decapitated at 2 or 6 h after IA injections was collected. Plasma samples were obtained by centrifugation and measured in duplicates. Plasma concentrations of OXT and AVP were determined by radioimmunoassay with specific anti-OXT and anti-AVP antibodies as described previously (38). Plasma concentrations of corticosterone (CORT) were measured with an ELISA kit (Corticosterone ELISA Kit, Cayman chem., MI, USA). The results were averaged for each group at each evaluation time (*n* = 6–7 per group and evaluation time).

### Statistical Analysis

All data are presented as mean ± standard error of the mean (SEM). Statistical analyses were conducted using two-way ANOVAs (experimental group as one and time after IA injection as second factor). Regarding the data of the changes in right knee transverse diameter and the mechanical nociceptive threshold, repeated measurements were done on the same set of experimental animals, therefore repeated measures ANOVAs were used. The two-way and repeated measures ANOVAs were followed, if applicable, by Bonferroni *post hoc* tests for multiple comparisons (Stata/IC 15; Stata Corp LP, TX, USA). *P*-values < 0.05 were considered statistically significant.

## Results

### Changes in Right Knee Transverse Diameter and Mechanical Nociceptive Threshold

**Figure 1:**
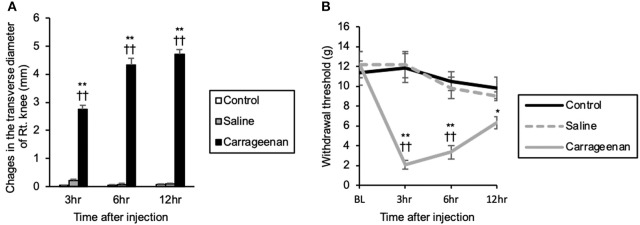
Changes in the transverse diameter of the ipsilateral knee and mechanical nociceptive threshold of the ipsilateral hind paw after intra-articular injection. The mean values of the changes in the transverse diameter of the ipsilateral knee measured by digital calipers are shown **(A)** (*n* = 6 per group). The mean values of the withdrawal thresholds to repetitive von Frey hair stimulation to the ipsilateral hind paw are shown **(B)** (*n* = 6 per group). The data are presented as mean ± standard error of the mean (Repeated measures ANOVA). *P < 0.05 and **P < 0.01 compared to control. ^†^^†^*P* < 0.01 compared to saline.

The repeated measures ANOVA showed that there was a statistically significant effect of experimental group and time after IA injection on the right knee transverse diameter [experimental group; *F*_(2, 39)_ = 1239.85, *P* < 0.01, time after IA injection; *F*_(2, 39)_ = 29.35, *P* < 0.01], and there was an interaction between experimental group and time after IA injection [*F*_(4, 39)_ = 34.14, *P* < 0.01]. Changes in the transverse diameter of the ipsilateral knee were significantly increased in the carrageenan group compared with those in the control and saline groups at 3 h (carrageenan vs. control; *P* < 0.01, carrageenan vs. saline; *P* < 0.01), 6 h (carrageenan vs. control; *P* < 0.01, carrageenan vs. saline; *P* < 0.01), and 12 h (carrageenan vs. control; *P* < 0.01, carrageenan vs. saline; *P* < 0.01) after injection (Figure 1A). Regarding the mechanical nociceptive threshold, there was a statistically significant effect of experimental group and time after IA injection on the mechanical nociceptive threshold [experimental group; *F*_(2, 60)_ = 28.62, *P* < 0.01, time after IA injection; *F*_(3, 60)_ = 8.97, *P* < 0.01], and there was an interaction between experimental group and time after IA injection [*F*_(6, 60)_ = 6.27, *P* < 0.01]. The mechanical nociceptive thresholds were significantly decreased in the carrageenan group compared with those in the control and saline groups at 3 h (carrageenan vs. control; *P* < 0.01, carrageenan vs. saline; *P* < 0.01) and 6 h (carrageenan vs. control; *P* < 0.01, carrageenan vs. saline; *P* < 0.01) after IA injection. However, the carrageenan-induced decrease in nociceptive threshold was gradually attenuated, and there was only a significant difference between the carrageenan and control groups 12 h after the IA injection (*P* < 0.05) (Figure 1B).

### The Expression of Fos-ir Cells in the L4 Dorsal Horn, the SON and PVN, and Colocalization With OXT and AVP-ir Neurons

**Figure 2:**
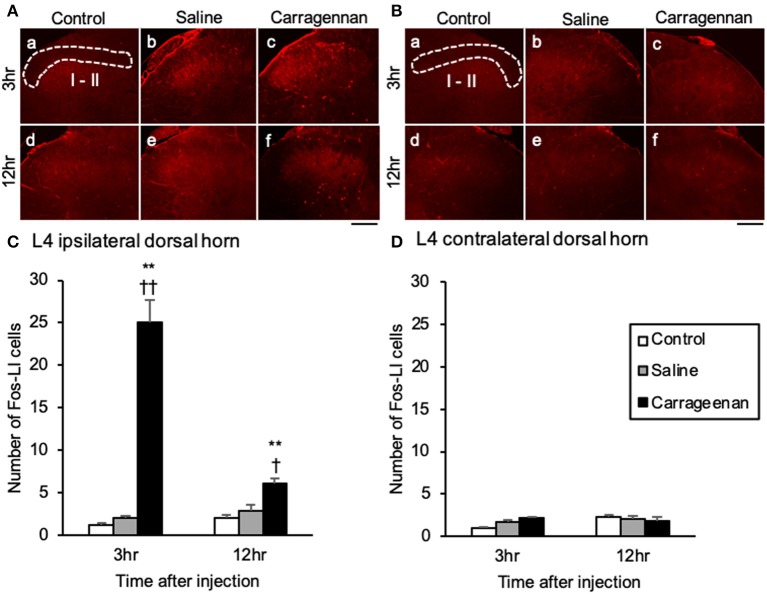
Expression of Fos-immunoreactive cells in lamina I and II of lumbar segment 4 dorsal horn after intra-articular injection. Fos-immunoreactive (ir) cells (red) in the ipsilateral lamina I and II **(A)** and contralateral side **(B)** are shown (*n* = 5–6 per group). Scale bars = 100 μm. The ipsilateral and contralateral lamina I and II are surrounded by white dotted lines (**A**-a, **B**-a). Numbers of Fos-ir cells in the ipsilateral lamina I and II **(C)** and contralateral side **(D)** are shown. The data are presented as mean ± standard error of the mean (two-way ANOVA). **P < 0.01 compared to control. ^†^*P* < 0.05 and ^†^^†^*P* < 0.01 compared to saline.

**Figure 3:**
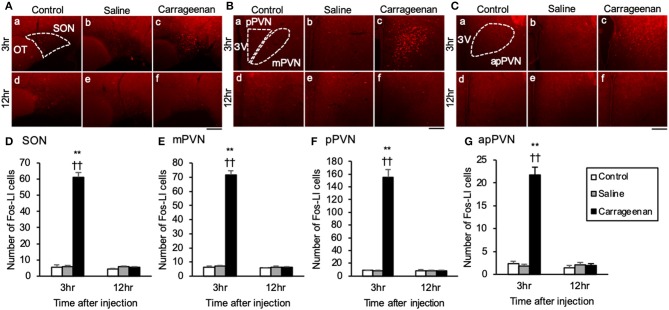
Expression of Fos-immunoreactive cells in the SON and PVN after intra-articular injection. Fos-immunoreactive (ir) cells (red) in the supraoptic nucleus (SON) **(A)**, magnocellular paraventricular nucleus (mPVN) **(B)**, parvocellular paraventricular nucleus (pPVN) **(B)**, and anterior parvocellular paraventricular nucleus (apPVN) **(C)** (*n* = 5–6 per group). Scale bars = 100 μm. The SON, mPVN, pPVN, and apPVN are surrounded by white dotted lines (**A**-a, **B**-a, **C**-a). The numbers of Fos-ir cells in the SON **(D)**, mPVN **(E)**, pPVN **(F)**, and apPVN **(G)** are shown. The data are presented as mean ± standard error of the mean (two-way ANOVA). **P < 0.01 compared to control. ^†^^†^*P* < 0.01 compared to saline.

For the number of Fos-immunoreactive (ir) cells in the ipsilateral (not contralateral) lamina I and II of the L4 dorsal horn, the SON, mPVN, pPVN, and apPVN, two-way ANOVA showed a statistical difference in experimental group [ipsilateral lamina I and II: *F*_(2, 26)_ = 64.42, *P* < 0.01, SON: *F*_(2, 26)_ = 237.09, *P* < 0.01, mPVN: *F*_(2, 26)_ = 362.20, *P* < 0.01, pPVN: *F*_(2, 26)_ = 103.64, *P* < 0.01, apPVN: *F*_(2, 26)_ = 87.15, *P* < 0.01] and time after IA injection [ipsilateral lamina I and II: *F*_(1, 26)_ = 12.48, *P* < 0.01, SON: *F*_(1, 26)_ = 235.89, *P* < 0.01, mPVN: *F*_(1, 26)_ = 358.15, *P* < 0.01, pPVN: *F*_(1, 26)_ = 98.64, *P* < 0.01, apPVN: *F*_(1, 26)_ = 87.65, *P* < 0.01]. The number of Fos- ir cells in lamina I and II of the L4 dorsal horn were significantly increased only on the ipsilateral side in the carrageenan group compared with those in the control and saline groups both at 3 h (carrageenan vs. control; *P* < 0.01, carrageenan vs. saline; *P* < 0.01) and 12 h (carrageenan vs. control; *P* < 0.01, carrageenan vs. saline; *P* < 0.05) after IA injection; however, the number of Fos-ir cells in the carrageenan group at 12 h after IA injection was substantially decreased compared with that at 3 h after IA injection [*F*_(1, 10)_ = 29.26, *P* < 0.01] (Figure 2). Further, the number of Fos-ir cells in the SON, mPVN, pPVN, and apPVN were significantly increased in the carrageenan group compared with those in the control and saline groups 3 h after the IA injection (SON: carrageenan vs. control; *P* < 0.01, carrageenan vs. saline; *P* < 0.01, mPVN: carrageenan vs. control; *P* < 0.01, carrageenan vs. saline; *P* < 0.01, pPVN: carrageenan vs. control; *P* < 0.01, carrageenan vs. saline; *P* < 0.01, apPVN: carrageenan vs. control; *P* < 0.01, carrageenan vs. saline; *P* < 0.01), but there was no significant difference 12 h after the IA injection (Figure 3).

**Figure 4:**
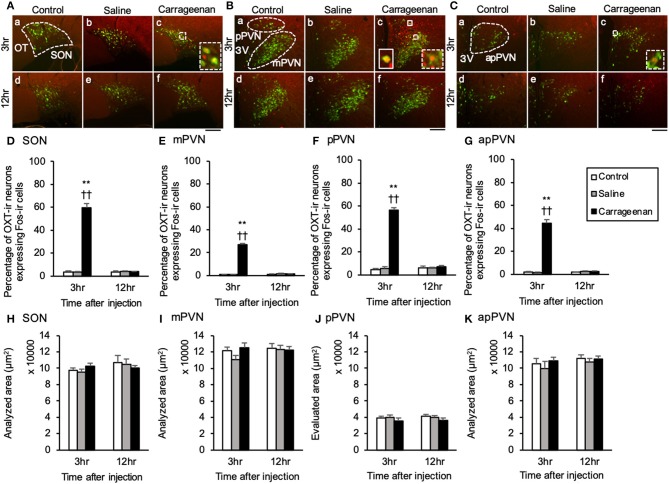
The percentage of oxytocin (OXT)-immunoreactive neurons expressing Fos-immunoreactivity in the SON and PVN after intra-articular injection. OXT-immunoreactive (ir) neurons (green) and Fos-ir cells (red) in the supraoptic nucleus (SON) **(A)**, magnocellular paraventricular nucleus (mPVN) **(B)**, parvocellular paraventricular nucleus (pPVN) **(B)**, and anterior parvocellular paraventricular nucleus (apPVN) **(C)** (*n* = 5–6 per group). Magnified images of OXT-ir expressing Fos immunoreactivity in the SON, mPVN, and apPVN are represented in the white square dotted lines (**A**-c, **B**-c, **C**-c). Magnified image of OXT-ir neurons expressing Fos immunoreactivity in the pPVN is represented in the white square lines (**B**-c). Scale bars = 100 μm. The SON, mPVN, pPVN, and apPVN are surrounded by white dotted lines (**A**-a, **B**-a, **C**-a). The percentages of OXT-ir neurons expressing Fos immunoreactivity in the SON **(D)**, mPVN **(E)**, pPVN **(F)**, and apPVN **(G)** are shown. The analyzed area for the SON **(H)**, mPVN **(I)**, pPVN **(J)**, and apPVN **(K)** are shown. The data are presented as mean ± standard error of the mean (two-way ANOVA). **P < 0.01 compared to control. ^†^^†^*P* < 0.01 compared to saline.

**Figure 5:**
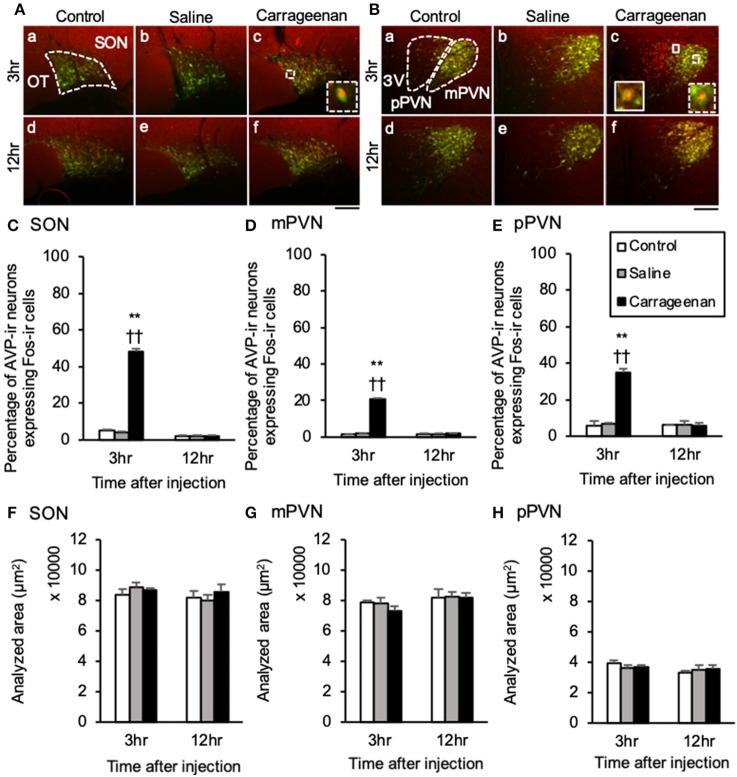
The percentage of arginine-vasopressin (AVP)-immunoreactive neurons expressing Fos-immunoreactivity in the SON and PVN after intra-articular injection. AVP-immunoreactive (ir) neurons (green) and Fos-ir cells (red) in the supraoptic nucleus (SON) **(A)**, magnocellular paraventricular nucleus (mPVN) **(B)**, and parvocellular paraventricular nucleus (pPVN) **(B)** are shown (*n* = 5–6 per group). Magnified images of AVP-ir neurons expressing Fos-immunoreactivity in the SON and mPVN are represented in the white square dotted lines (**A**-c, **B**-c). Magnified image of AVP-ir neuron expressing Fos-immunoreactivity in the pPVN is represented in the white square lines (**B-c**). Scale bars = 100 μm. The SON, mPVN, and pPVN are surrounded by white dotted lines (**A**-a, **B**-a). The percentages of AVP-ir neurons expressing Fos-immunoreactivity in the SON **(C)**, mPVN **(D)**, and pPVN **(E)** are shown. The analyzed area for the SON **(F)**, mPVN **(G)**, and pPVN **(H)** are shown. The data are presented as mean ± standard error of the mean (two-way ANOVA). **P < 0.01 compared to control. ^†^^†^*P* < 0.01 compared to saline.

For percentage of OXT-ir neurons expressing Fos-ir in the SON, mPVN, pPVN, and apPVN, two-way ANOVA showed a statistical difference in experimental group [SON: *F*_(2, 26)_ = 150.76, *P* < 0.01, mPVN: *F*_(2, 26)_ =3 35.89, *P* < 0.01, pPVN: *F*_(2, 26)_ = 220.37, *P* < 0.01, apPVN: *F*_(2, 26)_ = 167.72, *P* < 0.01] and time after IA injection [SON: *F*_(1, 26)_ = 139.92, *P* < 0.01, mPVN: *F*_(1, 26)_ = 284.45, *P* < 0.01, pPVN: *F*_(1, 26)_ = 168.94, *P* < 0.01, apPVN: *F*_(1, 26)_ = 139.42, *P* < 0.01]. The percentage of OXT-ir neurons expressing Fos-ir in the SON, mPVN, pPVN, and apPVN were significantly increased in the carrageenan group compared with those in the control and saline group 3 h after the IA injection (SON: carrageenan vs. control; *P* < 0.01, carrageenan vs. saline; *P* < 0.01, mPVN: carrageenan vs. control; *P* < 0.01, carrageenan vs. saline; *P* < 0.01, pPVN: carrageenan vs. control; *P* < 0.01, carrageenan vs. saline; *P* < 0.01, apPVN: carrageenan vs. control; *P* < 0.01, carrageenan vs. saline; *P* < 0.01) (Figure 4). For percentage of AVP-ir neurons expressing Fos-ir in the SON, mPVN and pPVN, two-way ANOVA showed a statistical difference in experimental group [SON: *F*_(2, 26)_ = 469.02, *P* < 0.01, mPVN: *F*_(2, 26)_ = 419.85, *P* < 0.01, pPVN: *F*_(2, 26)_ = 52.62, *P* < 0.01] and time after IA injection [SON: *F*(1, 26) = 610.10, *P* < 0.01, mPVN: *F*_(1, 26)_ = 388.23, *P* < 0.01, pPVN: *F*_(1, 26)_ = 52.30, *P* < 0.01]. The percentage of AVP-ir neurons expressing Fos-ir in the SON, mPVN, and pPVN were significantly increased in the carrageenan group compared with those in the control and saline groups at 3 h after the IA injection (SON: carrageenan vs. control; *P* < 0.01, carrageenan vs. saline; *P* < 0.01, mPVN: carrageenan vs. control; *P* < 0.01, carrageenan vs. saline; *P* < 0.01, pPVN: carrageenan vs. control; *P* < 0.01, carrageenan vs. saline; *P* < 0.01) (Figure 5). However, this percentage was smaller than that of OXT-ir neurons. All analyzed areas had no significant differences among any groups (Figures 4, 5).

### The Gene Expression of *OXT, CRH* mRNA and *AVP* hnRNA in the SON and PVN, and *POMC* mRNA in Anterior Pituitary

**Figure 6:**
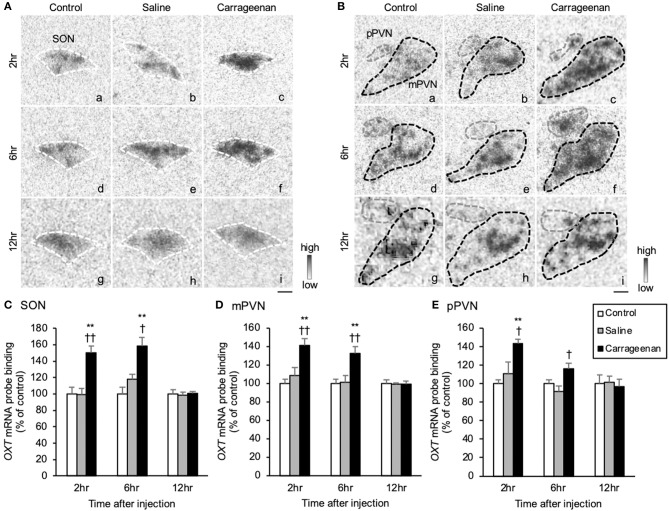
The expression of oxytocin (*OXT*) mRNA in the SON and PVN after intra-articular injection. The expression of *OXT* mRNA in the supraoptic nucleus (SON) **(A)**, magnocellular paraventricular nucleus (mPVN) **(B)**, and parvocellular paraventricular nucleus (pPVN) **(B)** are shown (*n* = 6–7 per group). The regions of interest for SON, mPVN, and pPVN in each group are surrounded by colored dotted lines as follows: SON, white **(A)**; mPVN. black; pPVN, gray **(B)**. Scale bars = 100 μm **(A,B)**. The *OXT* mRNA probe binding (% of control) in the SON **(C)**, mPVN **(D)**, and pPVN **(E)** are shown. The data are presented as mean ± standard error of the mean (two-way ANOVA). **P < 0.01 compared to control. ^†^*P* < 0.05 and ^†^^†^*P* < 0.01 compared to saline.

The gene expression levels of *OXT* mRNA in the SON, mPVN, and pPVN were significantly increased in the carrageenan group compared with those in the control and saline groups at both 2 and 6 h after IA injection (Figures 6A,B). For *OXT* mRNA probe binding level in the SON, mPVN, and pPVN, two-way ANOVA showed a statistical difference in experimental group [SON: *F*_(2, 48)_ = 22.74, *P* < 0.01, mPVN: *F*_(2, 48)_ = 15.18, *P* < 0.01, pPVN: *F*_(2, 48)_ = 7.84, *P* < 0.01] and time after IA injection [SON: no significant difference, mPVN: *F*_(2, 48)_ = 34.59, *P* < 0.01, pPVN: *F*_(2, 48)_ = 15.06, *P* < 0.01]. The *OXT* mRNA probe binding level was also significantly increased in the carrageenan group compared with that in the control and saline groups at both 2 h (SON: carrageenan vs. control; *P* < 0.01, carrageenan vs. saline; *P* < 0.01, mPVN: carrageenan vs. control; *P* < 0.01, carrageenan vs. saline; *P* < 0.01, pPVN: carrageenan vs. control; *P* < 0.01, carrageenan vs. saline; *P* < 0.05) and 6 h (SON: carrageenan vs. control; *P* < 0.01, carrageenan vs. saline; *P* < 0.05, mPVN: carrageenan vs. control; *P* < 0.05, carrageenan vs. saline; *P* < 0.05, pPVN: carrageenan vs. saline; *P* < 0.05) after IA injection. However, this change was attenuated to the same level as that for the control and saline groups 12 h after injection (Figures 6C–E).

**Figure 7:**
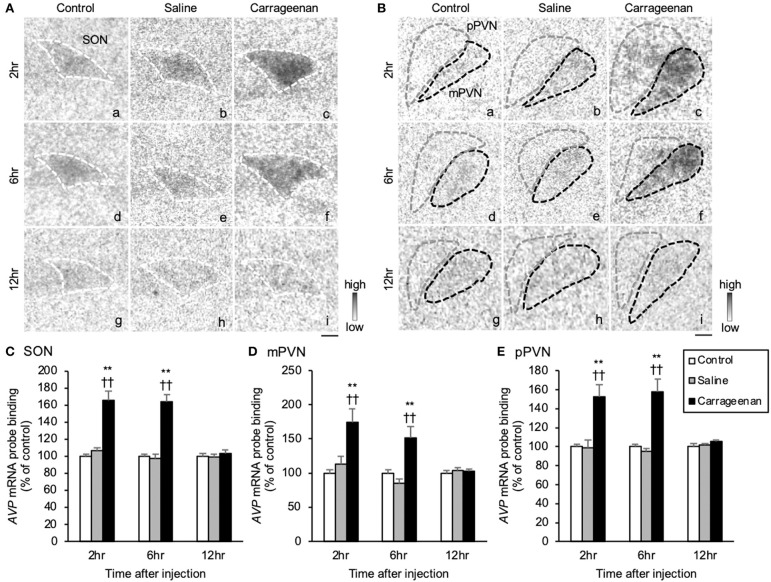
The expression of arginine-vasopressin (*AVP*) hnRNA in the SON and PVN after intra-articular injection. The expression of *AVP* hnRNA in the supraoptic nucleus (SON) **(A)**, magnocellular paraventricular nucleus (mPVN) **(B)**, and parvocellular paraventricular nucleus (pPVN) **(B)** (*n* = 6–7 per group). Scale bars = 100 μm **(A,B)**. The regions of interest for SON, mPVN, and pPVN in each group are surrounded by colored dotted lines as follows: SON, white **(A)**; mPVN, black; pPVN, gray **(B)**. The *AVP* hnRNA probe binding (% of control) in the SON **(C)**, mPVN **(D)**, and pPVN **(E)**. The data are presented as mean ± standard error of the mean (two-way ANOVA). **P < 0.01 compared to control. ^†^^†^*P* < 0.01 compared to saline.

The gene expression levels of *AVP* hnRNA in the SON, mPVN, and pPVN were significantly increased in the carrageenan group compared with that in the control and saline groups at both 2 and 6 h after IA injection (Figures 7A,B). For *AVP* hnRNA probe binding level in the SON, mPVN, and pPVN, two-way ANOVA showed a statistical difference in experimental group [SON: *F*_(2, 50)_ = 19.79, *P* < 0.01, mPVN: *F*_(2, 50)_ = 8.39, *P* < 0.01, pPVN: *F*(2, 50) = 20.27, *P* < 0.01] and time after IA injection [SON: *F*_(2, 50)_ = 119.05, *P* < 0.01, mPVN: *F*_(2, 50)_ = 138.34, *P* < 0.01, pPVN: *F*_(2, 50)_ = 376.76, *P* < 0.01]. The *AVP* hnRNA probe binding level was also significantly increased in the carrageenan group compared with that in the control and saline groups at both 2 h (SON: carrageenan vs. control; *P* < 0.01, carrageenan vs. saline; *P* < 0.01, mPVN: carrageenan vs. control; *P* < 0.01, carrageenan vs. saline; *P* < 0.01, pPVN: carrageenan vs. control; *P* < 0.01, carrageenan vs. saline; *P* < 0.01) and 6 h (SON: carrageenan vs. control; *P* < 0.01, carrageenan vs. saline; *P* < 0.01, mPVN: carrageenan vs. control; *P* < 0.01; carrageenan vs. saline; *P* < 0.01, pPVN: carrageenan vs. control; *P* < 0.01, carrageenan vs. saline; *P* < 0.01) after IA injection. However, this change was attenuated to the same level as that of the control and saline groups 12 h after the IA injection (Figures 7C–E).

**Figure 8:**
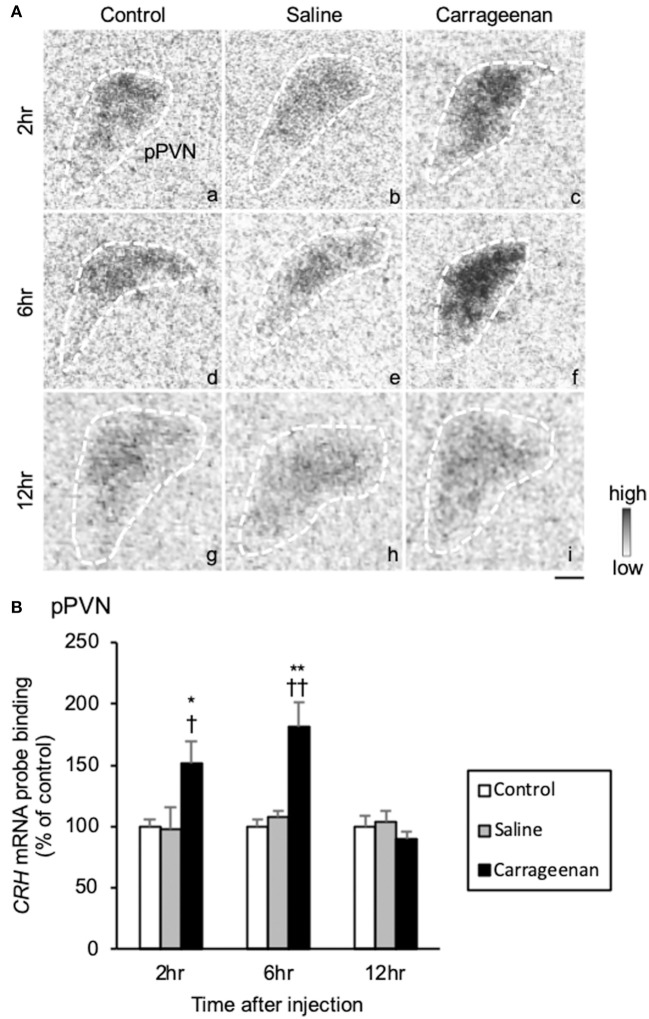
The expression of corticotropin releasing hormone (*CRH*) mRNA in the pPVN after intra-articular injection. The expression of *CRH* mRNA in the parvocellular paraventricular nucleus (pPVN) **(A)** is shown (*n* = 6–7 per group). The regions of interest for each group are surrounded by white dotted lines **(A)**. Scale bars = 100 μm **(A)**. The *CRH* mRNA probe binding (% of control) in the pPVN **(B)** is shown. The data are presented as mean ± standard error of the mean (two-way ANOVA). *P < 0.05 and **P < 0.01 compared to control. ^†^*P* < 0.05 and ^†^^†^*P* < 0.01 compared to saline.

Because the lack of the sensitive CRH antibody, we could not assess CRH neurological activity with FIHC. Therefore, CRH was only evaluated with regard to gene expression by ISH. The gene expression levels of *CRH* mRNA in the pPVN were significantly increased in carrageenan group compared with control and saline groups at both 2 and 6 h after IA injection (Figure 8A). For *CRH* mRNA probe binding level in the pPVN, two-way ANOVA showed a statistical difference in experimental group [*F*_(2, 49)_ = 13.48, *P* < 0.01) and time after IA injection [*F*_(2, 49)_ = 14.59, *P* < 0.01). The *CRH* mRNA probe biding level was also significantly increased in carrageenan group compared with control and saline groups at both 2 h (carrageenan vs. control; *P* < 0.05, carrageenan vs. saline; *P* < 0.05) and 6 h (carrageenan vs. control; *P* < 0.01, carrageenan vs. saline; *P* < 0.01) after injection. However, this change was attenuated to the same level as that of control and saline groups 12 h after IA injection (Figure 8B).

**Figure 9:**
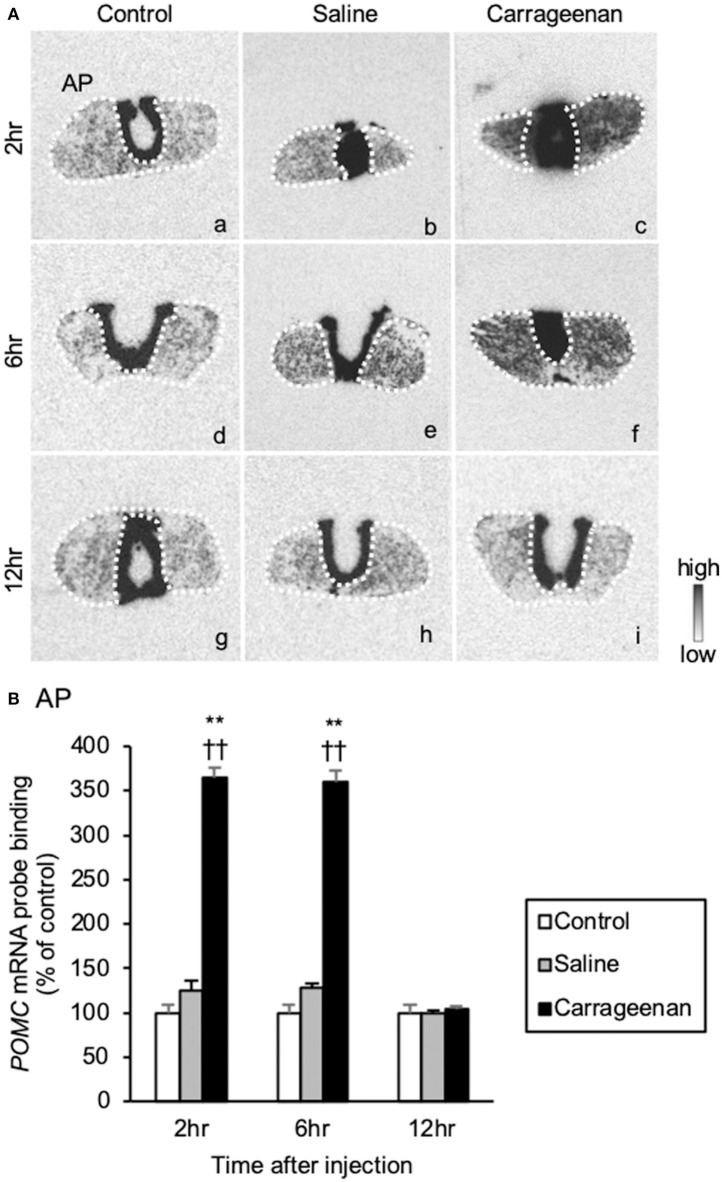
The expression of proopiomelanocortin (*POMC*) mRNA in the anterior pituitary after intra-articular injection. The expression of *POMC* mRNA in the anterior pituitary (AP) **(A)** is shown (*n* = 6–7 per group). The regions of interest of AP for each group are surrounded by white dotted lines **(A)**. Scale bars = 100 μm **(A)**. The *POMC* mRNA probe binding (% of control) in the AP **(B)** is shown. The data are presented as mean ± standard error of the mean (two-way ANOVA). **P < 0.01 compared to control. ^†^^†^*P* < 0.01 compared to saline.

The gene expression levels of *POMC* mRNA in the AP were significantly increased in the carrageenan group compared to those of control and saline groups at both 2 and 6 h after IA injection (Figure 9A). For *POMC* mRNA probe binding level in the AP, two-way ANOVA showed a statistical difference in experimental group [*F*_(2, 48)_ = 344.53, *P* < 0.01] and time after IA injection [*F*_(2, 48)_ = 30.51, *P* < 0.01]. The *POMC* mRNA probe binding level was also significantly increased in the carrageenan group compared with that of the control and saline groups at both 2 h (carrageenan vs. control; *P* < 0.01, carrageenan vs. saline; P < 0.01) and 6 h (carrageenan vs. control; *P* < 0.01, carrageenan vs. saline; *P* < 0.01) after IA injection. However, this change was attenuated to the same level as that of the control and saline groups at 12 h after IA injection (Figure 9B).

### Plasma OXT, AVP, and CORT

**Figure 10:**
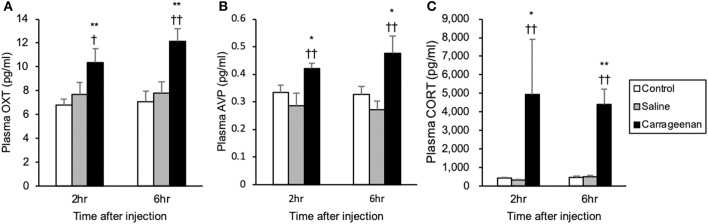
Plasma oxytocin (OXT), arginine-vasopressin (AVP), and corticosterone (CORT) after intra-articular injection. Plasma OXT **(A)**, AVP **(B)**, and CORT **(C)** concentrations measured with collected blood from decapitated rats are shown (*n* = 6–7 per group). The data are presented as mean ± standard error of the mean (two-way ANOVA). *P < 0.05 and **P < 0.01 compared to control. ^†^*P* < 0.05 and ^†^^†^*P* < 0.01 compared to saline.

For the plasma concentrations of OXT, AVP and CORT, two-way ANOVA showed a statistical difference in experimental group [OXT: *F*_(2, 32)_ = 25.17, *P* < 0.01, AVP: *F*_(2, 28)_ = 14.68, *P* < 0.01, CORT: *F*_(2, 30)_ = 18.14, *P* < 0.01]. The plasma concentrations of OXT, AVP, and CORT were significantly increased in the carrageenan group compared with those in the control and saline groups at both 2 h (OXT: carrageenan vs. control; *P* < 0.01, carrageenan vs. saline; *P* < 0.05, AVP: carrageenan vs. control; *P* < 0.05, carrageenan vs. saline; *P* < 0.01, CORT: carrageenan vs. control; *P* < 0.05, carrageenan vs. saline; *P* < 0.01) and 6 h (OXT: carrageenan vs. control; *P* < 0.01, carrageenan vs. saline; *P* < 0.01, AVP: carrageenan vs. control; *P* < 0.05, carrageenan vs. saline; *P* < 0.01, CORT: carrageenan vs. control; *P* < 0.01, carrageenan vs. saline; *P* < 0.01) after IA injection (Figure 10).

## Discussion

In the present study, we evaluated the effects of acute mono-arthritis on activation of the OXT/AVP system and HPA axis using a carrageenan-induced model of knee arthritis in rats. The acute mono-arthritic rats showed the significant increase in knee diameter and the significant decrease in the mechanical nociceptive threshold in the affected limb. IHC study revealed that the number of Fos-ir cells in the ipsilateral lamina I and II of the spinal dorsal horn, SON and PVN were significantly increased, and the percentage of OXT-ir and AVP-ir neurons expressing Fos-ir in both the SON and PVN were also increased in acute mono-arthritic rats. Further, ISH study revealed that levels of OXT mRNA and AVP hnRNA in the SON, mPVN and pPVN, CRH mRNA in the pPVN, and POMC mRNA in the AP were significantly increased in acute mono-arthritic rats. Further, plasma OXT, AVP, and CORT levels were significantly increased in acute mono-arthritic rats.

### Acute Mono-Arthritis Induced Hyperalgesia and Activated Neurons in the Ipsilateral Spinal Cord Lamina and Bilateral Hypothalamus

At first, acute mono-arthritis was confirmed by joint swelling and induced mechanical hyperalgesia. A previous study in rats reported that hyperalgesia of the ipsilateral hind paw developed in carrageenan-induced knee arthritis (39), and our result also showed that only carrageenan-injected rats exhibited increases in the ipsilateral knee diameter and mechanical hyperalgesia. To determine the time points for assessing neuronal activation in the spinal cord and hypothalamus caused by knee arthritis, we evaluated the progress of hyperalgesia after IA carrageenan injections. We found that hyperalgesia was induced 3 h after the carrageenan injection, and the mechanical nociceptive threshold was lowest at that timepoint. The decrease in the mechanical nociceptive threshold gradually recovered, similar to previous reports (39). Based on this result, we assumed that the OXT/AVP system was activated 3 h, but not 12 h, after the carrageenan IA injection, and decided to perform IHC at 3 and 12 h after the IA injection. Next, neuronal activation in the hypothalamus and spinal cord caused by acute mono-arthritis was evaluated by quantifying the number of Fos-ir cells. Fos protein is the metabolic product of the c-*fos* gene and is widely used as a marker of neuronal activity. It is considered useful for identifying brain areas activated by various physiological stimuli (40–42). Various types of peripheral stimulation of primary sensory fibers result in the expression of Fos protein in the nuclei of postsynaptic neurons of the dorsal horn of the spinal cord (43). Therefore, the mapping of Fos expression in these neurons is one of the best global markers for efficiently locating populations of neurons that respond to nociceptive input (44). For carrageenan-induced arthritis, numerous studies have used Fos expression in the spinal cord to evaluate the effectiveness of anti-inflammatory drugs and analgesics in a rat model of carrageenan-induced hyperalgesia/inflammation (45, 46). In the present study, Fos-ir cells in the lamina I-II in the dorsal horn (ipsilateral side only), SON, and PVN were significantly increased at 3 h after IA injection in rats with carrageenan-induced arthritis. These results were similar to those of a previous study which evaluated Fos-ir in the spinal cord, SON, and PVN after bilateral subcutaneous formalin injections into the hind paw (31, 34), but these studies assessed bilateral neuronal activation in lamina I-II, SON, and PVN. Our results revealed that neuronal activation in the spinal cord was induced only on the ipsilateral side of the administered IA carrageenan injection. Conversely, neuronal activation in the SON and PVN were induced in both ipsilateral and contralateral sides in rats with acute knee arthritis. This result suggested that acute mono-arthritis stimuli activated the ipsilateral nociceptive afferent pathway at the spinal level, as well as neurons in the hypothalamus which potentially modulate nociceptive pain, such as OXT, AVP, and CRH neurons.

### OXT/AVP System Activation Induced by Acute Mono-Arthritis

The neuronal activity specific to the OXT/AVP system was evaluated by assessing the percentage of OXT/AVP-ir neurons expressing Fos-ir by IHC and the gene expression of *OXT* mRNA and *AVP* hnRNA by ISH in the hypothalamus. The increase of the percentage of OXT-ir and AVP-ir neurons expressing Fos-ir at 3 h after the carrageenan IA injection were confirmed by IHC. As our previous study revealed that the gene expression of OXT mRNA and AVP hnRNA by ISH were increased earlier than the time point when those neurons co-express Fos-ir by IHC (8, 9), we decided to perform decapitation at 2 h after the carrageenan IA injection. However, it was unclear whether the plasma OXT and AVP were increased at that time point; therefore, we also performed decapitation at 6 h after the carrageenan IA injection. The result showed that gene expression levels of *OXT* mRNA and *AVP* hnRNA in the SON and PVN, as well as plasma OXT and AVP levels, were significantly increased in rats with acute knee arthritis at 3 and 6 h after the IA injection. These results were consistent with previous studies of various nociceptive models (8, 9, 31). OXT and AVP have been reported to produce local anti-nociception (47, 48). OXT and AVP neurons were activated, and their plasma concentrations were increased in models of formalin test and adjuvant-induced arthritis (7–10), but previous studies have only evaluated the activation of OXT and AVP neurons individually. Therefore, to our knowledge, this is the first study to report that the OXT/AVP system is simultaneously activated by nociceptive stimuli. Watanabe et al. (49) reported that transient receptor potential vanilloid type 1 (TRPV1) channels are involved in the development of carrageenan-induced mechanical hyperalgesia. TRPV1 channels are expressed in both peripheral and central nervous systems and play a crucial role in inflammatory pain (50, 51). Nersesyan et al. (52) reported that OXT acts as a partial agonist of TRPV1 channels and induces strong desensitization of these channels under nociceptive stimulation. Their study strongly suggests that at a peripheral level, OXT-induced analgesia passes at least in part through a direct TRPV1 interaction. Although the relationship between AVP and TRPV1 channels in nociception is unclear, Dayanithi et al. (53) reported a subpopulation of OXT and AVP-expressing dorsal root ganglion(DRG) neurons that express TRPV1 channels. The authors confirmed this subpopulation by demonstrating that most cultured DRG neurons responsive to capsaicin generated Ca^2+^ signals when challenged with OXT or AVP. These [Ca^2+^]i transients were blocked by specific agonists of OXT receptors and AVP V1 receptors, respectively. Their results suggested that OXT and AVP are expressed and released from sensory neurons to provide rapid and local analgesic effects via TRPV1 at the level of DRGs (53). Collectively, our results combined with those previous reports suggest that the OXT/AVP system mediates carrageenan-induced hyperalgesia via TRPV1 channels in rats with acute mono-arthritis.

### HPA Axis Activation Induced by Acute Mono-Arthritis

We conducted ISH to investigate activation of the HPA axis caused by acute mono-arthritis based on gene expression levels of HPA axis-related peptides including *AVP* hnRNA and *CRH* mRNA in the pPVN and *POMC* mRNA in the AP. ISH revealed that the expression levels of these genes were simultaneously increased by acute mono-arthritis concurrent with the increase of plasma CORT. AVP neurons in the pPVN and CRH neurons mediate HPA axis activation by stimulating adrenocorticotropic hormone (ACTH) secretion from the AP (1). ACTH secretion is initiated by POMC in the AP and promotes CORT secretion from the adrenal cortex (54–56). A previous study revealed that both AVP and CRH neurons were activated by acute nociceptive stimuli (9, 10). In contrast, chronic nociceptive stimuli, such as adjuvant arthritis, downregulate CRH neuronal activity; in these conditions, AVP neurons in the pPVN are more dominant and play crucial roles in HPA axis activation (10). In the present study, IHC and ISH analyses indicated that AVP neurons in the pPVN were activated, and the gene expression of *AVP* hnRNA in the pPVN was significantly increased in rats with acute knee arthritis. IHC revealed numerous Fos-ir cells in the pPVN which were not colocalized with either OXT-ir or AVP-ir. Although we were unable to evaluate these cells with IHC due to the low sensitivity of the CRH antibody, this result suggested that CRH neurons in the pPVN are activated by acute mono-arthritis. ISH indicated that expression levels of *CRH* mRNA were significantly increased in rats with acute knee arthritis. Further, expression levels of *POMC* mRNA in the AP and plasma levels of CORT were significantly increased in those rats. These results suggested that acute mono-arthritis activated both AVP neurons and CRH neurons in the pPVN associated with HPA axis activation, which differed from chronic multiple-arthritis.

This study has some limitations that need to be acknowledged. Although OXT and AVP may have anti-nociceptive effects on acute mono-arthritis, we did not conduct any pharmacological tests with OXT, AVP, or their antagonists. Therefore, the precise reasons for activation of the OXT/AVP system caused by acute mono-arthritis remains unclear. We plan to conduct further experiments to assess the potential analgesic effects of these hormones on arthritis.

In conclusion, the current study demonstrated that acute mono-arthritis activated ipsilateral nociceptive afferent pathways at the spinal level and simultaneously activated both the OXT/AVP system and HPA axis alongside upregulation of relevant gene expression in rats.

## Data Availability Statement

All datasets generated for this study are included in the article/supplementary material.

## Ethics Statement

The animal study was reviewed and approved by the Ethics Committee of Animal Care and Experimentation of the University of Occupational and Environmental Health, Japan.

## Author Contributions

MK and YU designed the research. HN, YM, KB, HU, SS, KN, KT, KS, and TO performed the research. YU and AS coordinated the study, participated in data collection, and wrote the paper together with HN, MK, TMat, HS, HO, YY, TF, MY, and TMar. All authors approved the final version of the manuscript and agreed to be accountable for all aspects of the work in ensuring that questions related to the accuracy. All authors designated as authors qualify for authorship, and all those who qualify for authorship are listed.

### Conflict of Interest

The authors declare that the research was conducted in the absence of any commercial or financial relationships that could be construed as a potential conflict of interest.
